# Engineered mesoporous silica nanosystems with organotin(iv) complexes containing 1-(quinolin-8-yliminomethyl)naphthalen-2-ol ligand for cancer cell targeting

**DOI:** 10.1039/d5dt02531a

**Published:** 2025-11-06

**Authors:** Diana Díaz-García, Robin Vinck, Javier Álvarez-Conde, Victoria García-Almodóvar, Sanjiv Prashar, Gilles Gasser, Santiago Gómez-Ruiz

**Affiliations:** a COMET-NANO Group, Departamento de Biología y Geología, Física y Química Inorgánica, ESCET, Universidad Rey Juan Carlos Calle Tulipán s/n E-28933 Móstoles Madrid Spain santiago.gomez@urjc.es; b Chimie ParisTech, PSL University, CNRS, Institute of Chemistry for Life and Health Sciences, Laboratory for Inorganic Chemical Biology 75005 Paris France gilles.gasser@chimieparistech.psl.eu; c Instituto de Investigación de Tecnologías para la Sostenibilidad, Universidad Rey Juan Carlos Calle Tulipán s/n E-28933 Móstoles Madrid Spain

## Abstract

The development of targeted nanotherapeutics has emerged as a promising approach to improve the efficacy and safety of anticancer treatments. This manuscript presents the synthesis, detailed characterization, and biological evaluation of novel mesoporous silica nanoparticles (sMSNs) functionalized with organotin(iv) complexes containing 1-(quinolin-8-yliminomethyl)naphthalen-2-ol ligand, which have also been selectively targeted using biotin (BT) and folic acid (FA). The systems have been comprehensively characterized using microscopy, nitrogen adsorption–desorption isotherms, and several spectroscopic methods confirming the successful conjugation of organotin(iv) complexes and targeting ligands, as well as the preservation of the structural integrity of the nanoparticles. The cytotoxic potential of the functionalized sMSNs was assessed *in vitro* using cancer cell lines (HeLa and MCF-7) and non-cancerous cell lines (Hek 293T and RPE-1) to evaluate their selectivity and biocompatibility. The results demonstrated that the tin-functionalized nanoparticles exhibited a significant antiproliferative activity. Notably, the incorporation of BIO and FA as targeting ligands enhanced selectivity toward cancer cells, minimizing toxicity in healthy cells. Our compounds were found to have a higher cytotoxic activity than cisplatin. These findings highlight the potential of tin-functionalized mesoporous silica nanoparticles as a robust platform for targeted cancer therapy, offering enhanced efficacy and reduced off-target effects compared to conventional platinum-based treatments.

## Abstract 2

The development of targeted nanotherapeutics has emerged as a promising approach to improve the efficacy and safety of anticancer treatments based on organotin(iv) compounds.

## Graphical abstract

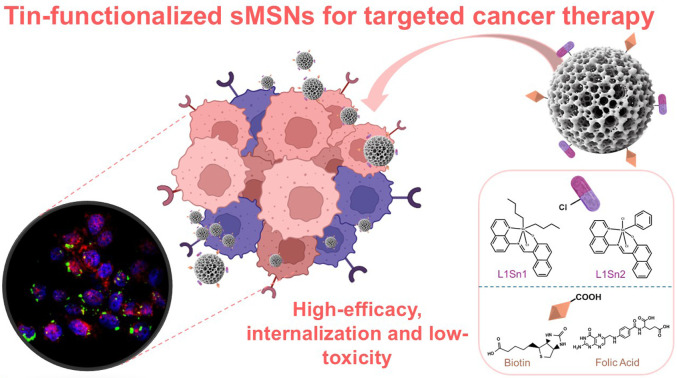

## 1. Introduction

Organotin(iv) complexes have garnered significant interest across diverse scientific fields due to their versatile coordination chemistry and ability to form stable bonds with a broad spectrum of ligands. Their notable applications include their role as catalysts in polymerization and hydrogenation reactions,^1^ as well as stabilizers in the synthesis of thermally stable and flame-retardant plastics and resins.^2^ Organotin(iv) compounds are also widely used in electrochemical systems, such as electrodes in batteries and electronic devices. However, in recent years, a burgeoning area of research has emerged around their potential as antitumor agents, marking a promising frontier in medicinal inorganic chemistry.^3,4^

Cancer remains the second leading cause of mortality worldwide, following cardiovascular diseases. Early detection and effective treatment are critical to preventing metastasis and improving patient outcomes.^5^ Among the available treatment modalities, chemotherapy remains a cornerstone of cancer management. Since the serendipitous discovery of the antiproliferative activity of cisplatin,^6^ platinum-based drugs have become the standard in chemotherapeutic regimens. It is believed that cisplatin forms highly stable adducts with guanine residues in DNA, leading to cell cycle arrest at the G2 phase and the upregulation of tumor suppressor proteins.^7^ Despite their efficacy, platinum-based drugs are associated with significant limitations, such as high cost, limited availability, and poor stability in biological media. Moreover, severe side effects and the development of drug resistance limit their long-term clinical use.^8^

In contrast, organotin(iv) complexes have emerged as promising alternatives due to their favorable pharmacological profiles. Since the 1980s, studies have reported the antitumor potential of various organotin(iv) derivatives,^9^ characterized by the general formula R_*n*_SnX_4−*n*_, where R represents organic ligands and X denotes halides or other anionic ligands. These complexes exhibit remarkable apoptotic induction in numerous cancer cell lines and possess notable advantages, such as improved selectivity, water solubility, and reduced toxicity compared to platinum-based drugs.^10^ Furthermore, unlike platinum compounds, organotin(iv) complexes do not induce resistance *via* P-glycoprotein-mediated efflux, as they are not substrates of this protein, thereby circumventing multidrug resistance mechanisms.^11,12^ However, the antitumor efficacy of these compounds is highly dependent on the nature and coordination geometry of the ligands bound to the tin center, highlighting the importance of ligand design in optimizing their biological activity.^3^ In recent years, Schiff base ligands and their organotin complexes have attracted considerable attention in coordination chemistry due to their straightforward synthesis, structural versatility, and notable stability. In particular, ligands bearing nitrogen, oxygen, and sulfur donor atoms are of significant interest because of their broad spectrum of biological activities, including antimicrobial, antifungal, and anticancer properties.^13^ Among the diverse family of Schiff bases, derivatives incorporating naphthalene moieties have demonstrated remarkable cytotoxic effects against various cancer cell lines, highlighting their potential as scaffolds for the development of metal-based therapeutic agents.^14^

Nanotechnology has played a pivotal role in enhancing the therapeutic potential of metal-based drugs by improving their stability, solubility, and selective delivery.^15,16^ Among the various nanomaterials investigated for biomedical applications, mesoporous silica nanoparticles (MSNs) have garnered significant attention due to their high surface area, tunable pore size, non-toxicity, biocompatibility, and ease of functionalization.^17,18^ The mesoporous structure facilitates the incorporation of therapeutic agents, while surface functionalization enables the addition of targeting ligands, enhancing selectivity and reducing off-target cytotoxicity. Moreover, MSNs can be engineered into multifunctional platforms capable of simultaneous drug delivery, photodynamic therapy, and diagnostic imaging, thereby paving the way for personalized and theranostic approaches to cancer treatment.^19,20^

In this context, previous research by our team has focused on designing diverse nanostructured platforms functionalized with a wide range of metal-based drugs. These platforms have demonstrated the ability to selectively target various cancer cell lines, as well as bacterial and parasitic pathogens.^21–30^

Building upon our previous research on the development of nanostructured platforms functionalized with various metal-based drugs for therapeutic applications, this study represents a significant step forward in the field since, by combining the intrinsic anticancer properties of organotin(iv) complexes with the biocompatible features of mesoporous silica, this study advances the field by introducing a robust nanotherapeutic platform overcoming key limitations in conventional chemotherapy, such as poor selectivity. Specifically, we report the synthesis and detailed characterization of novel MSNs functionalized with two distinct organotin(iv) complexes and further modified with biotin or folic acid as cancer-targeting moieties. Both biotin^31^ and folic acid^32^ are well-recognized for their high affinity toward overexpressed receptors found on the surface of various cancer cell types, thus enhancing the selectivity of our nanoplatforms.

Overall, our findings demonstrate that these organotin-functionalized MSNs exhibit remarkable cytotoxicity against cancer cells, while exhibiting minimal toxicity toward non-cancerous cell lines. The strategic incorporation of organotin(iv) complexes within the mesoporous silica framework not only improves their bioavailability and stability but also amplifies their therapeutic potential. This optimized design enhances the system's ability to protect the active compounds bringing them to cancer cells, thereby improving treatment precision. This work contributes to the ongoing development of next-generation nanomaterials for targeted cancer therapies, offering improved efficacy, reduced adverse effects, and the potential to minimize multidrug resistance.

## 2. Experimental

### 2.1. Reagents for the synthesis and cell lines

For the preparation of the silica nanoparticles tetraethyl orthosilicate (TEOS) and hexadecyltrimethylammonium bromide (CTAB) were purchased from Aldrich and triethylamine 99% (NEt_3_) from Acros Organics. The ligands 3-mercaptopropyltriethoxysilane (MP), and trimethoxysilyl-propyldiethylenetriamine (DETATMS) were purchased from Fluorochem. The reagents for EDAC coupling 1-ethyl-3-(3-dimethylaminopropyl)carbodiimide hydrochloride (EDAC). *N*-Hydroxysuccinimide (NHS) and folic acid (FA) were purchased from Aldrich and biotin (BIO) was purchased from TCI. For the synthesis of tin complexes, 2-hydroxy-1-naphthaldehyde, diphenyltin dichloride 96% and dibutyltin dichloride 96% were purchased from Aldrich and 8-aminoquinoline from Fluorochem. All the reagents were used without further treatment.

The cell lines used in this study were purchased from the American Type Culture Collection (ATCC, Manassas, VA, USA) and maintained according to the supplier's recommendations.

### 2.2. Characterization techniques

Transmission electron microscopy (TEM) was carried out with a JEOL JEM 1010, operating at 100 kV and a STEM JEOL F200 operating at 200 kV. The micrographs were analyzed using the program ImageJ.^33^ Dynamic Light Scattering (DLS) and *Z*-potential measurements were performed in a Litiziser 500 from Anton Paar, at pH 7.4 (PBS buffer) and culture medium (MEM supplemented with 10% fetal bovine serum). N_2_ gas adsorption–desorption isotherms were performed using a Micromeritics ASAP 2020 analyzer. Thermogravimetric analysis (TG) were obtained with a Shimadzu mod. DSC-50Q operating between 30 and 800 °C with a ramp of 20 °C min^−1^ and an intensity of 50 A under nitrogen. ICP-AES measurements were carried out in a Varian Vista AX Pro Varian 720-ES (*λ*_Sn_ = 283.998 nm). Ultraviolet–Visible Diffuse Reflectance (DR UV–Vis) graphics were obtained with a PerkinElmer LAMBDA 850+ UV/Vis Spectrophotometer equipment. ^1^H (400.13 MHz), ^13^C{^1^H} (100.52 MHz) and ^119^Sn (149 MHz) NMR spectra were recorded on a Bruker AVANCE DRX 400 spectrometer with CDCl_3_ as internal standard solvent and external standard tetramethylsilane (TMS). ^119^Sn MAS NMR spectra, were recorded on a Varian-Infinity Plus Spectrometer at 400 MHz for proton frequency (4 µs 90 pulse, 4000 transients, spinning speed of 6 MHz, contact time 3 ms, pulse delay 1.5 s). Fourier-transform infrared (FT-IR) spectra were obtained using a PerkinElmer Spectrum Two spectrophotometer (PerkinElmer, Waltham, Massachusetts, USA) over the 4000–400 cm^−1^ region, with samples prepared as KBr pellets. All the graphics were represented and processed with the software OriginPro 2016.^34^

### 2.3. Synthesis of the tin complex L1Sn1

**Scheme 1:**
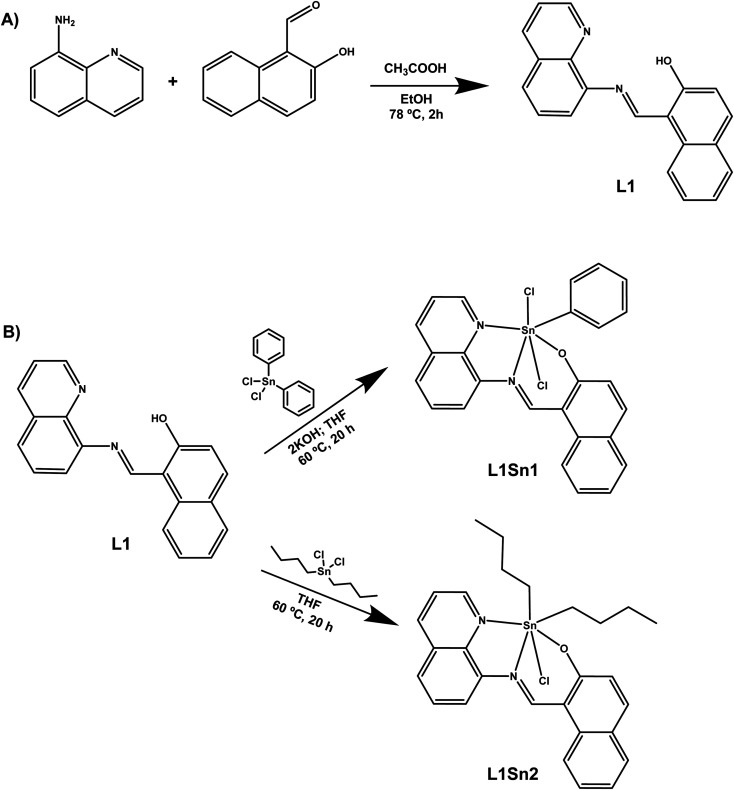
Synthetic procedure for the preparation of (A) ligand and (B) organotin compounds L1Sn1 and L1Sn2.

For the synthesis of the final complex L1Sn1, the ligand L1 was prepared (Scheme 1) as previously reported by Grusenmeyer *et al.* with minor modifications.^35^ For that, in a 100 mL Schlenk under nitrogen atmosphere quinolin-8-amine (288 mg, 1.99 mmol) and 2-hydroxy-1-naphthaldehyde (245 mg, 1.99 mmol) were added and dissolved in 20 mL of dry ethanol with a few drops of glacial acetic acid. The reaction was stirred at 110 °C for 2 hours. The reaction was cooled at room temperature, subsequently an orange precipitate appeared which was filtered, washed with MeOH and dried obtaining L1 as an orange solid with a 56% yield. ^1^H NMR (300 MHz, CDCl_3_) *δ*: 9.30 (d, *J* = 11.0 Hz, 1H), 9.08 (dd, *J* = 1.70 Hz, *J* = 4.20 Hz, 1H), 8.19 (dd, *J* = 1.70 Hz, *J* = 8.32 Hz, 1H), 8.00 (d, *J* = 8.32 Hz, 1H), 7.75 (dd, *J* = 1.28 Hz, *J* = 7.43 Hz, 1H), 7.70–7.45 (m, 6H), 7.29–7.25 (m, 1H), 6.91 (d, *J* = 9.45 Hz, 1H). Spectroscopic data were in accordance with the reported data (Fig. S1).

To obtain the organotin compound, L1 (50 mg, 0.17 mmol) was added in a Schlenk under a nitrogen atmosphere and dissolved in 10 mL of dried THF. Then a 2 M solution of KOH (0.167 mL, 0.33 mmol) was added and stirred for 15 minutes. Subsequently, a solution of diphenyl tin dichloride (57.6 mg, 0.17 mmol) in 10 mL of dried THF was transferred using a cannula to the reaction mixture and this mixture was then heated to 60 °C for 20 hours. Afterwards, the solvent was evaporated and washed with hexane to yield L1Sn1 as a brown solid. ^1^H NMR (300 MHz, CDCl_3_) *δ* (ppm): 9.30 (d, *J* = 11.02 Hz, 1H), 9.10–9.08 (m, 1H), 8.20 (dd, *J* = 1.43 Hz, *J* = 8.30 Hz, 1H), 8.01 (d, *J* = 8.20 Hz, 1H), 7.83–7.46 (m, 12H), 7.30–7.26 (m, 1H), 6.92 (d, *J* = 9.40 Hz, 1H). ^13^C{^1^H} NMR (101 MHz, CDCl_3_) *δ*: 181.9, 150.4, 147.6, 146.4, 140.1, 139.5, 137.8, 136.1, 134.4, 129.7, 129.1, 128.5, 127.5, 126.9, 126.8, 126.7, 124.5, 123.8, 122.5, 121.5, 118.5, 116.2, 113.3, 110.2, 109.0. ^119^Sn (300 MHz, CDCl_3_): −46.3. ^1^H NMR, ^13^C{^1^H} NMR and ^119^Sn spectra are given in the SI (Fig. S2, S3 and S4 respectively).

### 2.4. Synthesis of the tin complex L1Sn2

In a Schlenk under nitrogen a mixture of L1 (15 mg, 0.05 mmol) and dibutyl tin dichloride (15.2 mg, 0.05 mmol) was added under a nitrogen atmosphere and dissolved in 10 mL of dried THF. The reaction was heated at 60 °C overnight. The reaction was cooled at room temperature and the solvent was removed under vacuum; the resultant solid was washed with hexane to yield L1Sn2 as a brown solid. ^1^H NMR (300 MHz, CDCl_3_) *δ* (ppm): 9.35 (s, 1H), 9.10 (dd, *J* = 1.69 Hz, *J* = 4.23 Hz, 1H), 8.23 (dd, *J* = 1.69 Hz, *J* = 8.32 Hz, 1H), 8.03 (d, *J* = 8.30 Hz, 1H), 7.81–7.79 (m, 1H), 7.73–7.48 (m, 7H), 7.33–7.28 (m, 1H), 7.01 (d, *J* = 9.42 Hz, 1H), 1.82–1.78 (m, 8H), 1.45–1.39 (m, 4H), 0.95 (t, *J* = 7.31 Hz, 6H). ^13^C{^1^H} NMR (101 MHz, CDCl_3_) *δ*: 157.9, 150.5, 146.4, 145.1, 141.6, 141.0, 139.7, 136.1, 130.0, 129.8, 129.0, 128.4, 125.7, 124.4, 123.1, 119.2, 118.5, 117.9, 116.2, 113.3, 34.9, 28.1, 26.0, 14.0. ^119^Sn (300 MHz, CDCl_3_): 123.4. The ^1^H NMR, ^13^C{^1^H} NMR and ^119^Sn spectra are given in the SI (Fig. S5, S6 and S7 respectively).

### 2.5. Synthesis of small mesoporous silica nanoparticles (sMSN)

The synthesis of the sMSN material was carried out in accordance with two experimental procedures.^36,37^ Briefly, CTAB (1.0 g, 2.74 mmol, 1.5 equiv.) was dissolved in Milli-Q water (480 mL) in a 1 L round bottom flask. Then, triethylamine (251 µL, 1.80 mmol, 1 equiv.) was added, and the temperature increased to 80 °C. Once the temperature was reached, TEOS (5 mL, 22.40 mmol, 12.5 equiv.) was added dropwise under vigorous stirring (1500 rpm). The mixture was allowed to react for 3 hours at the same temperature. After that time, the reaction mixture was filtered and the solid thoroughly washed with water (2 × 500 mL) and methanol (1 × 100 mL). Finally, the solid was calcinated at 550 °C for 24 hours.

### 2.6. Functionalization with ligands

**Scheme 2:**
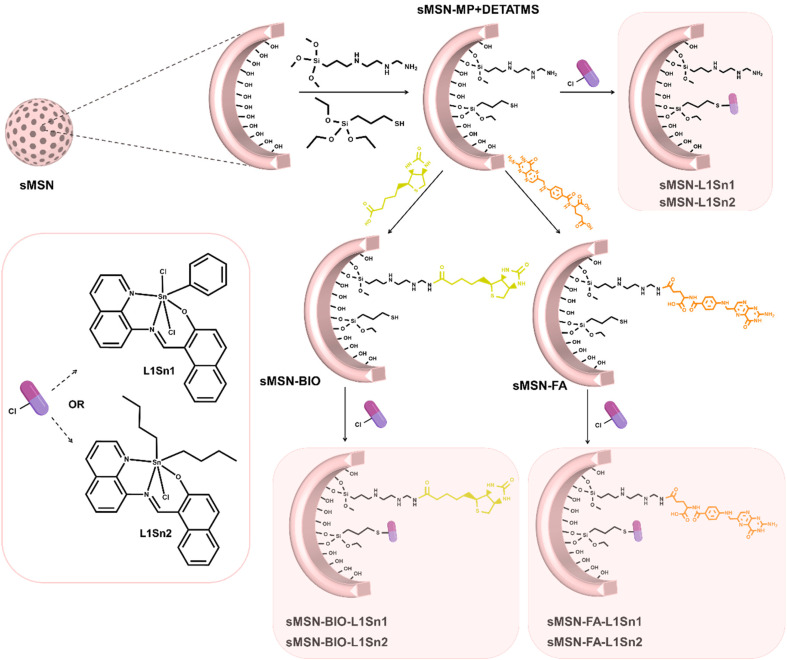
General procedure for the functionalization of silica-based materials.

The incorporation of the ligands 3-mercaptopropyltriethoxysilane (MP) and trimethoxysilyl-propyldiethylenetriamine (DETATMS) was carried out in a weight ratio 1 : 1 : 1 sMSN : MP : DETATMS. In brief, 2.0 g of sMSN was previously dried and activated under vacuum at 80 °C for 24 hours (Scheme 2). sMSN was then dispersed in dry toluene (50 mL) and MP (2.03 mL, 8.39 mmol) and DETATMS (1.94 mL, 7.53 mmol) were added under a nitrogen atmosphere. The mixture was stirred at 110 °C for 48 h. The functionalized-solid **sMSN-MP + DETATMS** was isolated by centrifugation (6000 rpm, 10 minutes), washed with toluene (2 × 25 mL) and diethyl ether (1 × 25 mL) and dried in a stove (75 °C).

### 2.7. Incorporation of targeting molecules

The synthesis of the materials with folic acid (FA) or biotin (BIO) was carried out by using an EDAC coupling reaction (Scheme 2). Briefly, 120 mg of FA or BIO (10% w/w) was dissolved in 30 mL of DMSO in ultrasonic bath and added to MES buffer pH 6.0 (120 mL) with EDAC (48 mg, 0.25 mmol) and NHS (78 mg, 0.68 mmol). After 15 minutes of carboxyl groups activation, 1.2 g of **sMSN-MP + DETATMS** were added, and the mixture was kept under vigorous stirring for 2 hours at room temperature. The materials sMSN-BIO or sMSN-FA were then isolated by centrifugation and washed with DMSO (2 × 15 mL) and ethanol (2 × 15 mL).

### 2.8. Incorporation of the tin complexes

For the preparation of the final materials with the tin complexes L1Sn1 or L1Sn2 (Scheme 2), 250 mg of the functionalized material **sMSN-MP + DETATMS**, sMSN-BIO or sMSN-FA were prepared in a Schlenk tube and subjected to two vacuum/nitrogen cycles (15 minutes/1 minute). The material was dispersed in dry toluene (10 mL) and NEt_3_ (22 µL, 0.16 mmol) was added to the dispersion. In another Schlenk tube, L1Sn1 or L1Sn2 (44 mg, 0.07 and 0.08 mmol respectively) were dissolved in dry toluene (10 mL) and added to the silica dispersion. The final mixture was heated at 60 °C for 24 hours. Subsequently, the solid was centrifugated and thoroughly washed with methanol (2 × 10 mL) and water (1 × 10 mL) and dried in a stove. The final products were referenced as sMSN-L1Sn1, sMSN-BIO-L1Sn1, sMSN-FA-L1Sn1, sMSN-L1Sn2, sMSN-BIO-L1Sn2 and sMSN-FA-L1Sn2.

### 2.9. General remarks on biological studies and cell culture

#### 2.9.1. Release study

A study was conducted to analyze the release of Sn(iv) soluble species in simulated body fluid using phosphate-buffered saline (PBS; pH 7.4). In this experiment, 5 mg of the synthesized materials (sMSN-FA-L1Sn1, sMSN-FA-L1Sn2, sMSN-BIO-L1Sn1, and sMSN-BIO-L1Sn2) were dispersed in 5 mL of buffer solution and then incubated at 37 °C under agitation at 30 rpm in a Roto-Therm incubator (Benchmark Scientific, Sayreville, NJ, USA) for durations of 6 and 24 hours and 7 days. Following each incubation period, the suspensions were filtered through a 0.2 μm nylon filter and the resulting solutions were analyzed in duplicate using inductively coupled plasma atomic emission spectroscopy (ICP-AES) to quantify the amount of Sn(iv) soluble species leached into the solution. The same procedure was carried out with the final materials sMSN-FA-L1Sn1 and sMSN-FA-L1Sn2 incubating them in a more realistic medium with albumin from bovine serum, BSA (Aldrich) and l-glutathione reduced, Glu (Alfa Aesar) at a concentration of 1 mg mL^−1^ of each molecule in PBS, for 7 days at 37 °C. The same experiments were also carried out in PBS adjusted to pH 5.5 using the same materials and conditions.

#### 2.9.2. Cell culture

Human cervical carcinoma cells (HeLa) and human embryonic kidney (Hek 293T) cells were cultivated in MEM medium supplemented with 10% fetal bovine serum. Breast cancer cells (MCF-7) were cultivated in DMEM and human retinal pigment epithelial cells (RPE-1) in DMEM/F12 supplemented with 10% fetal bovine serum. All the media were complemented with penicillin–streptomycin mixture and incubated under a humidified atmosphere containing 5% CO_2_ at 37 °C.

#### 2.9.3. *In vitro* cytotoxicity assay

The cytotoxicity of the materials was tested using a fluorometric assay. Cells were seeded in 96-well plates (4000 cells per well in 100 μL of media). After 24 hours of incubation, the medium was removed, and the cells were treated with increasing concentrations of the materials in cell media achieving a total volume of 200 μL. The serial dilutions of each material were prepared in medium from a concentration of 0.01 µM to 100 µM in each well. The free tin complexes and cisplatin stock solutions were first prepared by dissolving the compounds in 200 µL of DMSO or water at a concentration of 10 000 µM. From these, dilutions were made with culture medium to reach a working concentration of 10 µM After 48 hours of incubation, the medium was removed, a resazurin dilution in medium (0.2 mg mL^−1^ in 100 μL) was added to the wells and the plates were incubated for 4 hours. The fluorescence of resorufin was read (excitation/emission 540/590 nm) using a SpectraMax M2 Microplate Reader (Molecular Devices). Fluorescence data were normalized and IC_50_ were calculated using GraphPad Prism software.^38^

#### 2.9.4. Confocal study

80 000 cells of MCF-7 per well were seeded in a 6-well plate and incubated under standard conditions. After the incubation period, the culture medium was replaced with fresh medium containing the final materials sMSN-L1Sn2, sMSN-BIO-L1Sn2 and sMSN-FA-L1Sn2, functionalized with 5% (w/w) of fluorescein isothiocyanate (FITC) (by EDAC coupling following the same protocol as for biotin and folic acid incorporation), at a concentration of 50 µM, prepared as previously described. Following exposure to the materials, the medium was removed, and cells were washed with PBS. Subsequently, the cells were treated sequentially with 10% formamide for 15 minutes, wheat germ agglutinin (WGA, 5 µM) for 10 minutes, and DAPI (1 µM) for 10 minutes. For confocal microscopy imaging, an Olympus Fluoview FV3000 microscope was used. Finally, the cells were mounted using Mowiol mounting medium for confocal microscopy imaging.

#### 2.9.5. Cytometry assay

A total of 2 × 10^6^ cells of MCF-7 were incubated with the final material sMSN-L1Sn2, sMSN-BIO-L1Sn2 and sMSN-FA-L1Sn2. Then the cells were collected and centrifuged at 1800 rpm for 5 minutes. The supernatant was carefully discarded, and the pellet was gently resuspended in 500 µL of 70% ethanol pre-chilled at −20 °C. The suspension was gently agitated for 2 minutes and then centrifuged at 5000 rpm for 5 minutes. The pellet was resuspended in 1 mL of PBS, centrifuged again at 5000 rpm for 5 minutes, and the supernatant was removed. The resulting pellet was resuspended in 900 µL of PBS. Next, 10 µL of RNase (10 mg mL^−1^ stock; final concentration: 100 µg mL^−1^) and 60 µL of propidium iodide (0.05% stock solution; final concentration: 0.003%) were added to the suspension. The mixture was gently agitated and incubated at 37 °C for 30 minutes with constant shaking. Finally, the samples were analyzed using flow cytometry (FACS Melody Becton Dickinson and CytoFLEX Beckman Coulter).

#### 2.9.6. Internalization assay

For the cellular internalization assays, 8 × 10^5^ cells per well of Hek 293T and MCF-7 were incubated with each nanomaterial at a concentration of 100 μM in culture medium for 24 hours. After the incubation period, the cells were harvested and subjected to digestion with 70% nitric acid (HNO_3_) for an additional 24 hours to ensure complete dissolution of the nanomaterials prior to quantification by ICP-AES.

## 3. Results and discussion

**Fig. 1:**
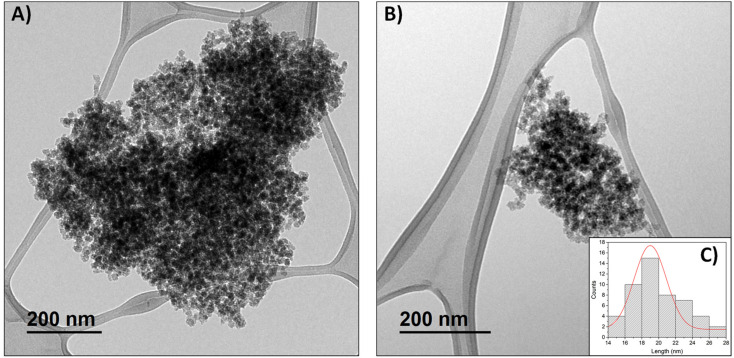
TEM micrographs of starting silica material (A and B) and histogram of particle size (C).

A mesoporous silica-based material (sMSN) was synthetized starting from the surfactant CTAB in an aqueous medium basified with triethylamine and using TEOS as silicon source and silica precursor. TEM analysis of the obtained sMSN showed a very small particle size around 20 ± 3 nm with a spherical morphology. Fig. 1 and Fig. S8 (SI) show the homogeneous distribution of the nanoparticles, which tend to form aggregates in solid state, likely due to the grid preparation process for TEM measurement. Nevertheless, the porosity of the silica is evident here and also in other micrographs of functionalized sMSN (Fig. S9, SI), which demonstrate that the morphology and size of these silicas remain unaffected by the successive functionalization steps.

The textural properties of sMSN were analyzed by nitrogen gas adsorption–desorption, using the BET method (Table 1). The starting material shows a large superficial area (*ca.* 766 m^2^ g^−1^) and interesting porosity comparable to other mesoporous silicas with internal ordering.^39,40^ After the incorporation of the organotin complex L1Sn1, the surface area decreased up to 4 times in comparison with the empty silica particles, demonstrating the incorporation of the complex on both the surface of the material and inside the pores due also to the considerable decrease in pore volume. The functionalization with the targeting molecules BIO or FA also resulted in a reduction of the superficial area and pore volume in comparison with the non-functionalized material sMSN-L1Sn1 which confirmed its functionalization.

**Table 1:** Textural parameters obtained by nitrogen adsorption/desorption isotherms

Material	*S*_BET_ (m^2^ g^−1^)	*V*_p_ (cm^3^ g^−1^)	*D*_p_ (nm)
sMSN	766	1.9	10.2
sMSN-L1Sn1	190	0.6	13.0
sMSN-L1Sn2	192	0.7	14.3
sMSN-BIO-L1Sn1	115	0.4	14.0
sMSN-BIO-L1Sn2	219	0.8	14.6
sMSN-FA-L1Sn1	90	0.3	14.6
sMSN-FA-L1Sn2	223	0.8	13.9

To further assess the dispersion behavior and hydrodynamic size of the materials under physiological-like conditions, we performed Dynamic Light Scattering (DLS) measurements in both PBS and DMEM culture medium. As shown in Fig. S10, the starting silica nanoparticles (sMSN) display an average hydrodynamic diameter of approximately 80 nm in PBS, indicating minor agglomeration that is not considered significant. In DMEM (Fig. S11A), the DLS data reveal two size populations, one centered around 60 nm and another around 90 nm. To clarify the origin of the 60 nm peak, we also performed a DLS measurement of the DMEM medium alone (Fig. S11B), which showed a similar signal around 60 nm, corresponding to protein aggregates naturally present in the culture medium. Therefore, we attribute the 90 nm population observed in Fig. S11A to the actual size distribution of the sMSN particles in DMEM.

Zeta potential measurements of three final materials were carried out to assess the surface charge and colloidal stability of the synthesized mesoporous silica nanosystems in the same two media PBS and DMEM (Table S1). The results show a clear influence of the surrounding medium and surface functionalization on the nanoparticle surface properties. In PBS, all three materials exhibited slightly negative zeta potential values, ranging from −2.2 to −4.4 mV. These low absolute values suggest limited electrostatic repulsion between particles and point to a tendency for mild agglomeration in saline buffer, which is consistent with the DLS data shown in Fig. S10. However, the mesoporous silica core and dense surface modification with organotin complexes and targeting ligands may contribute to steric stabilization, preventing severe aggregation under these conditions. In contrast, when dispersed in DMEM medium, a more complex environment that includes serum proteins and various ions, a notable shift in zeta potential was observed. For sMSN-L1Sn2 and sMSN-BIO-L1Sn2, the surface charge became more negative, likely due to the adsorption of negatively charged proteins, such as albumin, forming a protein corona. This moderate increase in surface charge may enhance colloidal stability in biological media through improved electrostatic and steric repulsion. Interestingly, sMSN-FA-L1Sn2 showed an almost neutral zeta potential in DMEM which could be attributed to charge screening effects or extensive protein adsorption on the nanoparticle surface. Despite this near-neutral charge, the DLS analysis (Fig. S11A) did not show significant aggregation, suggesting that other stabilization mechanisms, such as steric hindrance from surface-bound folic acid or interactions with the protein corona, are sufficient to maintain colloidal dispersion in this medium.

**Fig. 2:**
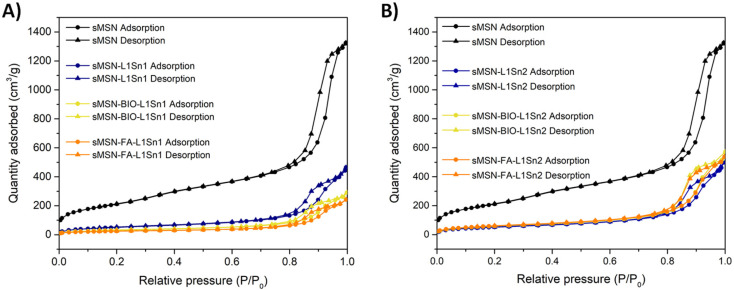
N_2_ isotherms of starting silica (sMSN) and the final materials with L1Sn1 (A) and L1Sn2 (B).

The BET analysis of adsorption isotherms for sMSN and some of the final materials were recorded in Fig. 2.

In all cases type IV isotherms with a hysteresis loop between types H_1_ and H_2_ was observed. The loop is observed at high pressures (*P*/*P*_0_ between approximately 0.75 and 0.90), which is characteristic of mesoporous systems and results from the pore-filling process *via* capillary condensation. As *P*/*P*_0_ increases, a continuous transition occurs from multilayer adsorption to complete pore filling. The hysteresis loops indicate, therefore, narrow pore distributions, with cylindrical morphology and uniform particle distribution as in other ordered silica-based mesoporous materials.^41^

**Fig. 3:**
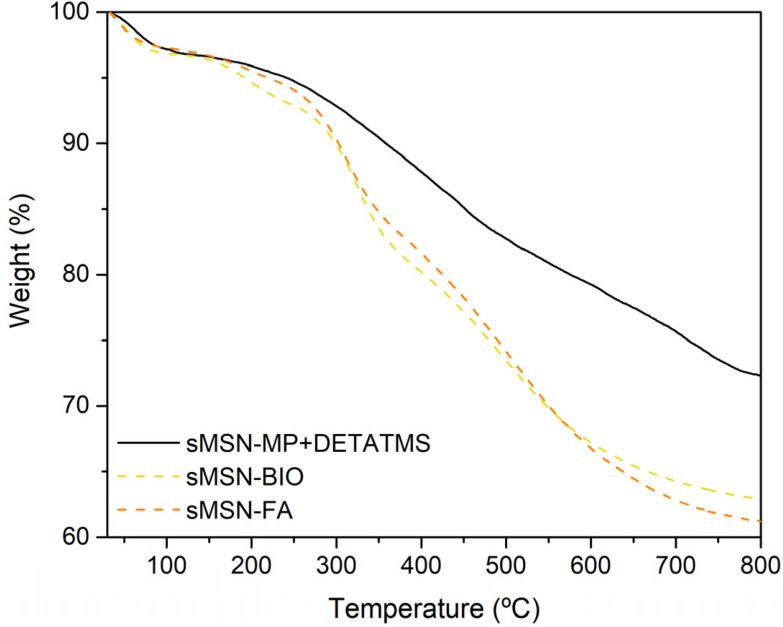
Thermogravimetric curves of some nanomaterials functionalized with different ligands.

For the quantification of the incorporation of the ligands MP and DETATMS and the targeting molecules biotin and folic acid, a thermogravimetric study was carried out. Regarding the loss of organic load for each material in Fig. 3, the functionalization of the ligands was 23.6% in weight taking into account the weight loss between 125 °C and 750 °C (to exclude deviations coming from the weight assigned to volatiles and silica condensation). This quantity of incorporation is in agreement with other silica materials functionalized with the same ligands.^42^ The functionalization ratio with the targeting molecules was practically identical, with a percentage of 5.9% for biotin and 6.7% for folic acid considering the temperature range between 125–750 °C.

The amount of tin covalently attached to the silica nanoparticles was quantified using ICP-AES. The results presented in Table 2 indicate a tin incorporation of 0.4–1.2 wt% in the final materials, corresponding to 2.1–6.2 wt% of the tin complexes, which translates to an encapsulation yield of 14–41%. These values represent the loading capacity/grafting efficiency of the organotin(iv) complexes on the sMSNs. Comparing the different materials, it is observed that functionalization with targeting molecules did not significantly affect the incorporation of tin complexes. However, folic acid functionalization resulted in a slightly higher yield compared to biotin, possibly due to the presence of two available carboxylic groups on folic acid for binding of organotin moieties.

**Table 2:** Percentages of tin and tin compound of all functionalized silicas obtained by ICP-AES

Material	%Sn	%Sn compound
sMSN-L1Sn1	1.570	8.01
sMSN-L1Sn2	0.988	4.71
sMSN-FA-L1Sn1	1.207	6.16
sMSN-FA-L1Sn2	0.875	4.17
sMSN-BIO-L1Sn1	0.735	3.75
sMSN-BIO-L1Sn2	0.440	2.10

**Fig. 4:**
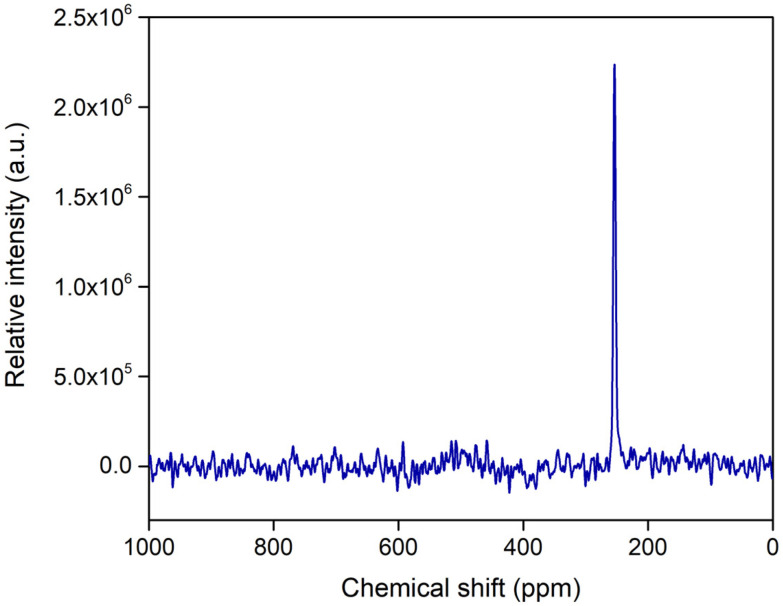
^119^Sn MAS NMR spectrum of the final material sMSN-L1Sn2.

The ^119^Sn MAS NMR spectrum of sMSN-L1Sn2 (Fig. 4) was recorded to confirm the incorporation of the tin complex to the system. The spectrum showed a unique intense signal around 250 ppm that corresponds to the covalent binding of the tin complex with the mercapto ligand (S–Sn bond). The absence of additional signals indicates that there is only a single species of the covalently incorporated metal complex onto the silica. The spectrum of sMSN-BIO-L1Sn1 shows a similar signal, although due to the coupling with the rest of the functionalization of the system, this spectrum shows a higher background noise (Fig. S12).

**Fig. 5:**
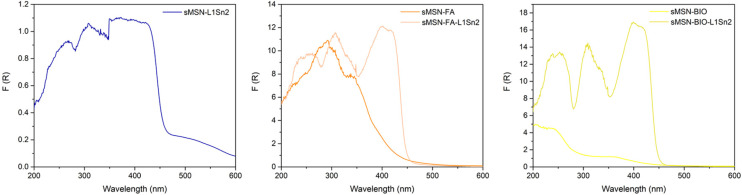
Diffuse reflectance UV-vis spectra of some functionalized materials.

All the materials were also characterized by diffuse UV-visible spectroscopy to determine their incorporation in the silica nanoparticles (Fig. 5).

The free tin compounds show a very broad spectrum between 350–450 nm (Fig. S13). A similar band was also observed in the silica nanoparticles, thus further demonstrating their successful incorporation in the nanomaterial. The successful functionalization with the targeting molecules (FA and/or BIO) is also confirmed by the appearance of a strong absorption band at 265 nm in the case of sMSN-FA or at *ca.* 240 nm for sMSN-BIO.

FT-IR spectrometry was used to confirm the functionalization and incorporation of different moieties through the formation of new covalent bonds. The obtained spectra show characteristic vibration bands of the attached compounds, allowing comparison with those of the pristine nanoparticles and confirming the incorporation of biologically relevant molecules (Fig. S15).

For the starting MSN, typical bands at 3400–3500, 1100, and 800 cm^−1^ correspond to –OH (Si–OH), Si–O–Si, and Si–OH vibrations, with the first also attributed to residual water (Fig. S14). After functionalization, new bands around 3400 and 3000 cm^−1^ appear, associated with free amines and C–H vibrations due to new covalent bonds. Upon BIO or FA conjugation, additional bands at 1650 and 2900 cm^−1^ arise from C=O and N–H groups and the EDAC-mediated amide linkage between surface amines and carboxyl groups. In Sn-containing materials, a new band at 750 cm^−1^ indicates Sn–S bond formation (Fig. S15).

**Fig. 6:**
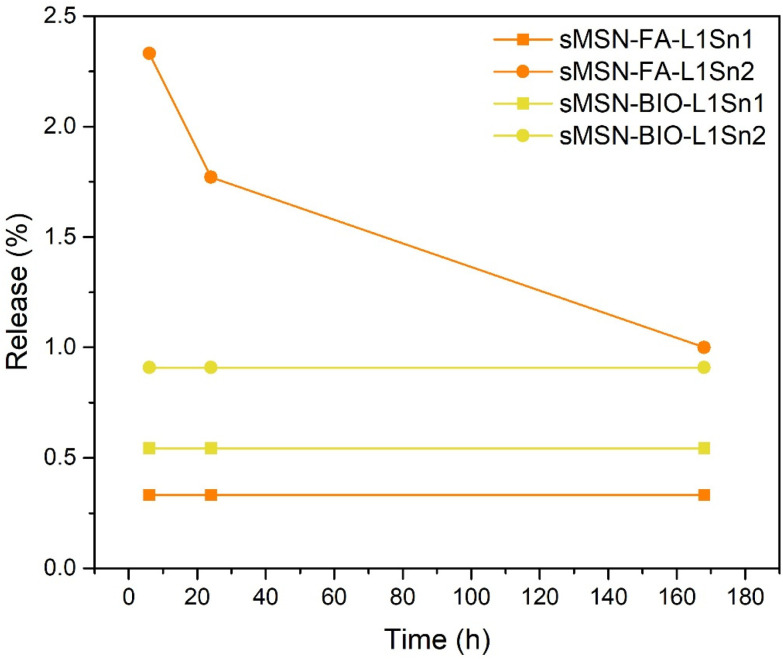
Tin release profile of the final materials to the biological medium at 6 and 24 hours and 7 days of incubation.

To confirm that the metal complexes are strongly bound to the silica *via* covalent bonds, a tin release study was conducted under simulated biological conditions (PBS buffer, pH 7.4, 37 °C) at different incubation times. As shown in Fig. 6, the percentage of metal released into the medium did not exceed 2.5% relative to the amount of tin complex incorporated into the nanomaterials. This result indicates that the biological activity of these tin-loaded materials arises from the action of the nanosystem as a whole, rather than from the release of metal species from the silica, as the tin is covalently bound. Even after prolonged incubation (up to 7 days), no degradation of the silica or significant release of tin into the medium was observed, demonstrating the stability of the designed systems. Additionally, a release experiment was performed with sMSN-FA-L1Sn1 and sMSN-FA-L1Sn2 in a more physiologically relevant medium containing excess BSA and glutathione to assess whether these molecules facilitate the release of metal complexes. As shown in Table S2 (SI), the release of tin into the medium, relative to the total functionalized amount in the material, is slightly higher in the presence of excess albumin and glutathione. However, this increase is not significant enough to suggest an albumin- or glutathione-induced tin release. This behavior indicates the covalent binding of the Sn species, because if the tin compound were merely adsorbed onto the silica-based nanomaterial, rather than covalently bound, a much higher release would be expected. Consequently, the therapeutic action of these materials does not seem to result from the release of metal species at the target site but rather from the nanomaterials acting as a whole, protecting the therapeutic Sn complexes during transport and ensuring their delivery to the target zone.

The anticancer therapeutic potential of the synthesized materials was evaluated using a fluorometric assay after 48 hours of incubation of the nanoparticles or free tin compounds with cancerous cell lines exhibiting different expression profiles of the biotin receptor (BR) or folic acid receptor alpha (FRA). Human breast cancer cells (MCF-7, BR+), human cervical carcinoma cells (HeLa, BR+, FRA+) were used to assess the effect of the targeting groups on the selectivity of the nanoparticles for the different cell lines. Human retinal pigment epithelial cells (RPE-1) and human embryonic kidney cells (Hek 293T) were included as a non-cancerous control cell lines.^43–46^

As shown in Table 3, the starting nanovehicle (sMSN) exhibited no toxicity in any of the tested cell lines, demonstrating the high biocompatibility of the unmodified silica system. While the activity of the tin-functionalized nanomaterials may initially appear similar to or lower than that of the free tin complexes, recalculating the IC_50_ values based on the amount of tin incorporated into each material (values provided in brackets) reveals a different trend. Specifically, the nanomaterials functionalized with L1Sn2 show greater activity than the free tin complex. For instance, in the case of MCF-7 cells, the tin-functionalized silica materials exhibited activities 3 to 8 times higher than those of the free L1Sn2 complex.

**Table 3:** Cytotoxic activity in different cell lines as a function of metal content and standard deviation

Material	IC_50_ (µM)			
	RPE-1	MCF-7	HeLa	Hek 293T
sMSN	>100	>100	>100	>100
sMSN-L1Sn1	0.22 ± 0.04	0.56 ± 0.04	1.18 ± 0.16	1.00 ± 0.16
sMSN-FA-L1Sn1	0.12 ± 0.01	>1.21 ± 0.00	>1.21 ± 0.00	>1.21 ± 0.00
sMSN-BIO-L1Sn1	0.07 ± 0.01	>0.74 ± 0.00	>0.74 ± 0.00	>0.74 ± 0.00
sMSN-L1Sn2	0.05 ± 0.01	0.04 ± 0.00	0.07 ± 0.01	0.02 ± 0.0
sMSN-FA-L1Sn2	0.07 ± 0.01	0.09 ± 0.02	0.10 ± 0.01	0.07 ± 0.01
sMSN-BIO-L1Sn2	0.04 ± 0.00	0.06 ± 0.01	0.02 ± 0.01	0.02 ± 0.01
L1Sn1	0.35 ± 0.06	0.25 ± 0.02	0.16 ± 0.02	0.25 ± 0.08
L1Sn2	0.44 ± 0.06	0.31 ± 0.04	0.23 ± 0.19	0.27 ± 0.06
**Cisplatin**	10.37 ± 2.98	10.43 ± 2.59	10.95 ± 0.06	5.18 ± 1.94

However, this trend does not apply to the L1Sn1 complex, as all the final materials functionalized with this complex show higher IC_50_ values than the free tin complex, indicating lower anticancer activity. This may be due to the higher incorporation of targeting molecules, such as biotin and folic acid, as confirmed during final material characterization using the BET technique. The results showed a 7-fold and 9-fold decrease in surface area for the sMSN-BIO-L1Sn1 and sMSN-FA-L1Sn1 materials, respectively, compared to the starting silica (sMSN), along with a reduction in pore volume (pore capping). These findings suggest that although the goal of incorporating biotin and folic acid was to increase the activity of the nanomaterials by improving their selectivity, the excess of these biomolecules may have unexpectedly promoted cell survival. This effect is particularly noticeable in cell lines that overexpress biotin receptors, such as MCF-7 and HeLa, likely due to biotin's role in supporting cellular metabolism.^47^ In this context, and considering the very low tin release (Fig. 6) for all the studied materials, the presence of targeting molecules such as biotin or folic acid seem to certainly play an important role in the cytotoxic nature of the materials.

Despite these results, the anticancer activity of the new tin complexes, both in their isolated form and when supported on silica nanoparticles, is remarkable compared to the well-known chemotherapy drug cisplatin. As shown in Table 3, the tin complexes, particularly the L1Sn2-functionalized materials, exhibit significantly higher activity than cisplatin. For example, against the MCF-7 breast cancer cell line, the tin-based materials are 116 to 268 times more potent, while for the HeLa cervical carcinoma cell line, their activity is between 110 and 547 times higher.

These findings highlight the promising potential of tin-based complexes loaded onto silica-based nanomaterials in chemotherapy, offering significant advantages over conventional platinum-based drugs. Additionally, the comparison of cytotoxic activity between cancerous and healthy cell lines shows that the toxicity of the tin-functionalized materials in non-cancerous cells is not elevated, remaining similar or lower compared to cancer cells. This selective toxicity is a crucial advantage for real-world applications, as it suggests that these materials could provide effective cancer treatment with reduced side effects.

**Fig. 7:**
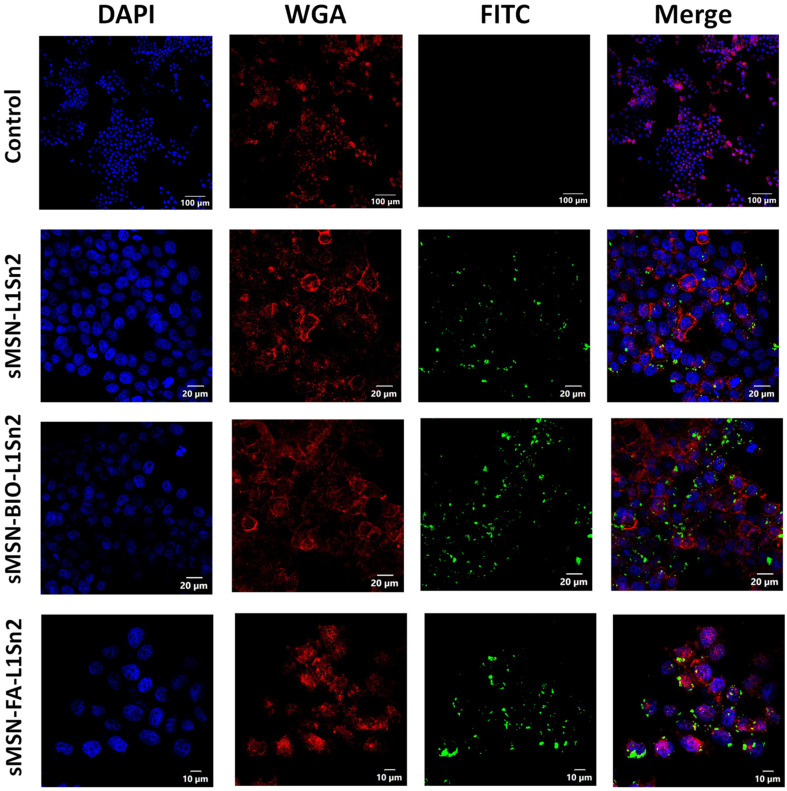
Confocal microscopy images of MCF-7 cells after incubation with the tin-functionalized nanomaterials (50 µM) for 24 h. The control corresponds to untreated cells. The nuclei were stained with DAPI (blue), the cell membranes with wheat germ agglutinin (WGA, red), and the nanoparticles were visualized through their FITC signal (green). The images show the internalization and intracellular distribution of the nanomaterials compared to the control sample.

Confocal microscopy was used to evaluate the cellular uptake and localization of three different final mesoporous silica in MCF-7 cells. As expected in the control, no green fluorescence was detected, confirming the absence of autofluorescence or nonspecific signal from the imaging system. Cell membranes and nuclei appeared well-defined, demonstrating normal morphology and integrity (Fig. 7).

Cells were treated with sMSN-L1Sn2 and a clear green fluorescence signal was observed, mainly localized near the cell membrane and within the cytoplasm. This indicates successful cellular uptake, most likely *via* passive internalization or nonspecific endocytosis. However, the signal was relatively sparse and less intense compared to the functionalized systems. The third row shows cells treated with sMSN-BIO-L1Sn2. In this case, the green fluorescence signal was more prominent and densely distributed throughout the cytoplasm. The increased signal intensity and number of internalized particles suggest enhanced uptake due to biotin-receptor-mediated endocytosis, as biotin receptors are overexpressed in MCF-7 cells. The merged images showed strong colocalization with membrane and cytoplasmic regions, confirming intracellular trafficking. Finally, the cells treated with the material sMSN-FA-L1Sn2 showed the highest green fluorescence intensity, with widespread nanoparticle accumulation throughout the cytoplasm and near the perinuclear region. This strong internalization is consistent with folate receptor-mediated uptake, as MCF-7 cells also overexpress folate receptors. The high degree of colocalization between the FITC signal and the red membrane stain supports the efficient receptor targeting and internalization of these nanosystems.

**Fig. 8:**
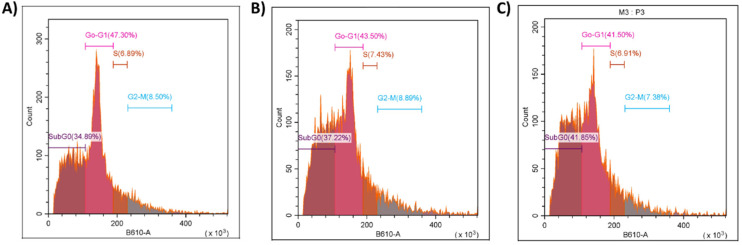
Histograms of flow cytometry analysis of MCF-7 cells treated with sMSN-L1Sn2 (A), sMSN-BIO-L1Sn2 (B) and sMSN-FA-L1Sn2 (C). A total of 2 × 10^6^ cells were used for each experiment. After treatment, cells were collected by centrifugation at 1800 rpm for 5 min, resuspended in cold ethanol (−20 °C), and gently vortexed for 2 min. The suspension was centrifuged again for 5 min, and the resulting pellet was washed with PBS. Subsequently, cells were incubated in a PBS/RNase solution (1 : 9 v/v) containing propidium iodide for 30 min at 37 °C under gentle agitation. The fluorescence intensity corresponding to propidium iodide was then recorded to assess cell cycle distribution.

Flow cytometry analysis was performed to evaluate the effect of the different functionalized mesoporous silica nanoparticles on the cell cycle distribution of treated cancer cells (Fig. 8). A prominent accumulation of cells in the Sub-G0 phase was observed in all treatments, indicating the induction of apoptosis. Notably, the percentage of cells in Sub-G0 increased from 34.89% in sMSN-L1Sn2 (A) to 37.22% in sMSN-BIO-L1Sn2 (B) and reached the highest value of 41.85% in the folic acid-functionalized system (C). This progressive increase suggests that both biotin and folic acid targeting enhance the pro-apoptotic activity of the organotin(iv)-based nanosystems, with folic acid showing the most significant effect. The proportion of cells in the G0–G1 phase slightly decreased across the treatments: 47.30% in condition (A), 43.50% in (B), and 41.50% in (C). This reduction correlates with the increase in the Sub-G0 population, implying that a portion of the cells exiting the G0–G1 phase undergo apoptotic death.

In contrast, the percentages of cells in the S and G2-M phases remained relatively constant among the three formulations. The S phase ranged from 6.89% to 7.43%, while the G2-M phase fluctuated between 7.38% and 8.89%. These values indicate that the materials did not seem to induce a specific arrest in DNA synthesis or mitosis, and their primary mode of action appears to be the induction of apoptosis rather than cell cycle arrest.

The internalization of the functionalized nanomaterials was evaluated in healthy (Hek 293T) and tumor (MCF-7) cells using ICP-AES (Table 4). Overall, a higher uptake of the nanomaterials was observed in tumor cells, consistent with their functionalization with active targeting compounds. In Hek 293T cells, the nanomaterial containing only the ligand L1 and the metal complex (sMSN-L1Sn2) showed a relative internalization of 0.74%, whereas functionalization with BIO and FA significantly increased cellular uptake, reaching values of 1.63% and 1.53%, respectively. On the other hand, in MCF-7 cells, a general increase in internalization was observed for all materials. The pristine system (sMSN-L1Sn2) reached a value of 1.02%, while the nanomaterials functionalized with BIO and FA showed higher values (1.81% and 1.71%, respectively).

**Table 4:** Cellular internalization of the materials determined by ICP-AES. The experiments were performed using Hek 293T and MCF-7 cells seeded at a density of 8 × 10^5^ cells per well. The cells were incubated with the different tin-functionalized materials (100 µM) for 24 h. After incubation, the cells were digested with 70% nitric acid, and the elemental content was quantified

Material	%Sn internalization Hek 293T	%Sn internalization MCF-7
sMSN-L1Sn2	0.74 ± 0.04	1.02 ± 0.05
sMSN-FA-L1Sn2	1.53 ± 0.06	1.71 ± 0.07
sMSN-BIO-L1Sn2	1.63 ± 0.03	1.81 ± 0.04

These results confirm that BIO and FA promote the cellular uptake of nanomaterials, with a more pronounced effect in tumor cells. This behavior can be explained by the overexpression of BIO and folate receptors in MCF-7 cells, which facilitates receptor-mediated internalization and enhances intracellular accumulation of the nanomaterials. This behavior had been previously observed in studies conducted by our group, where an increase in internalization of the materials in tumor cells was noted when they were functionalized with FA.^30,39^

## 4. Conclusions

This study focuses on the synthesis and characterization of sMSNs functionalized with two distinct tin complexes (L1Sn1 and L1Sn2) and biological targeting molecules, such as BIO or FA, to enhance selectivity toward specific cancer cells. The characterization of the functionalized nanoparticles was performed using various techniques to assess their structural and chemical properties. Nitrogen adsorption–desorption BET analysis confirmed that the initial sMSNs had a large surface area and high porosity. The successful incorporation of the tin complexes and subsequent functionalization with BIO or FA were verified through FTIR and NMR spectroscopy, as well as a measured decrease in surface area and pore volume, indicating the presence of these components within the nanoparticles. TEM studies further revealed that the small nanoparticles maintained a uniform size and morphology, even after the incorporation of the tin complexes.

Tin release studies under simulated biological conditions demonstrated that the nanoparticles released less than 2.5% of the encapsulated tin, highlighting the high stability of the system. Cytotoxicity evaluations using fluorometric assays in cancerous (MCF-7 and HeLa) and non-cancerous (Hek 293T and RPE-1) cell lines showed that the L1Sn2-functionalized nanoparticles exhibited significantly higher antitumor activity compared to both the L1Sn1-functionalized system and cisplatin, a widely used chemotherapy drug. Notably, the L1Sn2-functionalized nanoparticles were, in some cases, up to 268 times more effective compared to cisplatin. Importantly, the toxicity in healthy cells remained comparable to or lower than that observed for the free tin complexes or cisplatin, indicating a favorable safety profile.

These findings underscore the exceptional potential of tin-functionalized sMSN as selective and potent antitumor agents. The results suggest that these systems may provide superior efficacy and lower toxicity than conventional platinum-based drugs, such as cisplatin, due to their selective targeting of cancer cells and reduced impact on healthy cells provided that the functionalization with the selective agents is improved since most of the FA and BIO molecules are probably contained in the pore of the particles, which prevents their interaction with the cellular FA and BIO receptors. Further studies could aim to selectively functionalize the surface of the particle, which could help to achieve some selectivity towards cancer cells. Confocal analysis and flow cytometry studies support the hypothesis that folic acid and biotin functionalization enhance the cellular uptake and apoptotic efficacy of the organotin(iv)-loaded mesoporous silica nanoparticles. Among them, the folate-targeted system (sMSN-FA-L1Sn2) demonstrated the strongest pro-apoptotic effect, likely due to the interaction with folate receptors overexpressed on the surface of cancer cells.

In conclusion, this work provides evidence that tin-functionalized mesoporous silica nanoparticles, represent a promising platform for the development of more effective and less toxic anticancer therapies. Future studies focusing on optimizing the functionalization with targeting molecules could further enhance the therapeutic potential of these nanomaterials, opening new avenues for innovation in oncology treatments.

## Conflicts of interest

The authors whose names are listed immediately above certify that they have no affiliations with or involvement in any organization or entity with any financial interest (such as honoraria; educational grants; participation in speakers’ bureaus; membership, employment, consultancies, stock ownership, or other equity interest; and expert testimony or patent-licensing arrangements), or non-financial interest (such as personal or professional relationships, affiliations, knowledge or beliefs) in the subject matter or materials discussed in this manuscript.

## Supplementary Material

DT-054-D5DT02531A-s001

## Data Availability

The data supporting this article have been included as part of the supplementary information (SI). Supplementary information contains: NMR spectra of the compounds, additional TEM and STEM images of the materials, DLS study of the systems, *Z*-potential measurements, solid-state NMR spectra, DR-UV visible spectra of the materials, FTIR study of the materials and comparative tin release determination. See DOI: https://doi.org/10.1039/d5dt02531a.
