# Supplementary material for: Engineered mesoporous silica nanosystems with organotin(iv) complexes containing 1-(quinolin-8-yliminomethyl)naphthalen-2-ol ligand for cancer cell targeting
Source: Dalton Trans. 2025 Nov 6;54(47):17564–77. doi: 10.1039/d5dt02531a (PMC12608062; doi:10.1039/d5dt02531a)
Supplement: DT-054-D5DT02531A-s001 [file DT-054-D5DT02531A-s001.pdf]

Supplementary Information (SI) for Dalton Transactions.
This journal is © The Royal Society of Chemistry 2025

SUPPLEMENTARY MATERIAL

Engineered Mesoporous Silica Nanosystems with Organotin(IV) Complexes Containing 1-(quinolin-8-yliminomethyl)naphthalen-2-ol Ligand for Cancer Cell Targeting

Diana Díaz-García,[a,b] Robin Vinck,[b] Javier Álvarez Conde,[a] Victoria García-Almodóvar,[a] Sanjiv Prashar,[a,c] Gilles Gasser,[b,*] and Santiago Gómez-Ruiz[a,c**]

[a] *COMET-NANO Group, Departamento de Biología y Geología, Física y Química Inorgánica, ESCET, Universidad Rey Juan Carlos, Calle Tulipán s/n, E-28933, Móstoles (Madrid), Spain.*

[b] *Chimie ParisTech, PSL University, CNRS, Institute of Chemistry for Life and Health Sciences, Laboratory for Inorganic Chemical Biology, 75005 Paris, France.*

[c] *Instituto de Investigación de Tecnologías para la Sostenibilidad, Universidad Rey Juan Carlos, Calle Tulipán s/n, E-28933, Móstoles (Madrid), Spain*

This supplementary material contains:

1) ^{1}H NMR spectra of **L1** (Figure S1)
2) ^{1}H NMR spectra of **L1Sn1** (Figure S2)
3) ^{13}C{^{1}H} NMR spectra of **L1Sn1** (Figure S3)
4) ^{119}Sn spectra of **L1Sn1** (Figure S4)
5) ^{1}H NMR spectra of **L1Sn2** (Figure S5)
6) ^{13}C{^{1}H} NMR spectra of **L1Sn2** (Figure S6)
7) ^{119}Sn spectra of **L1Sn2** (Figure S7)
8) Additional TEM images of **sMSN** (Figure S8)
9) STEM images of **sMSN** and **sMSN-FA-L1Sn1** (Figure S9)
10) Particle size of **sMSN** in PBS by dynamic light scattering (Figure S10)
11) Particle size of **sMSN** in DMEM by dynamic light scattering (Figure S11)
12) Z- potential measurements for some final materials (Table S1)
13) ^{119}Sn MAS NMR spectrum of **sMSN-L1Sn1** (Figure S12)
14) DR UV-visible spectra of **L1Sn1** and **L1Sn2** (Figure S13)
15) FT-IR spectra of the starting material (sMSN) and final materials (Figure S14 and S5)
16) Comparative tin release in different incubation media (Table S2)

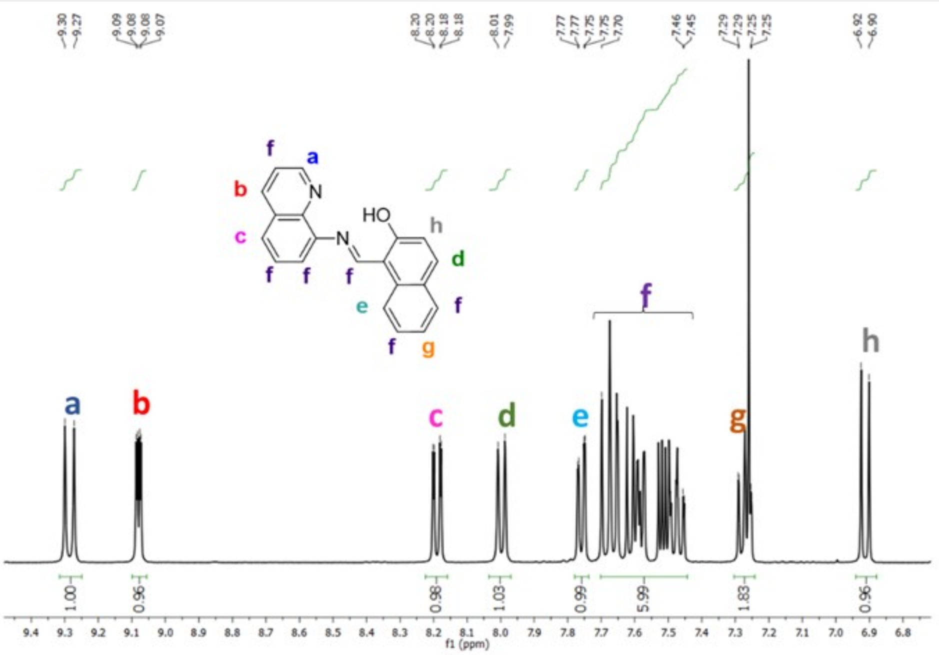

***Figure S1**. ^{1}H NMR spectra of the ligand **L1**.*

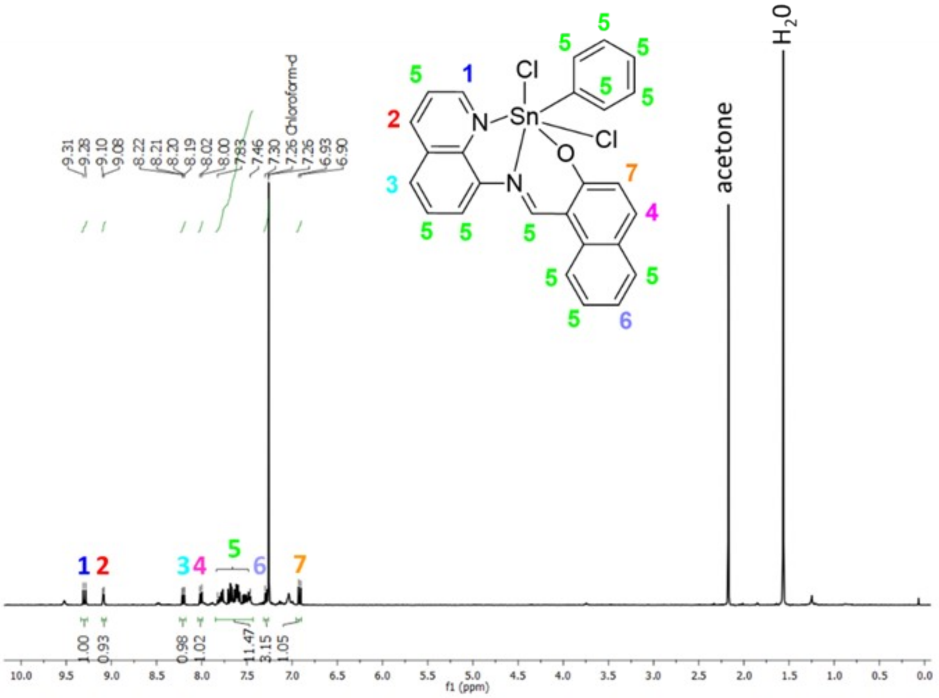

***Figure S2**. ^{1}H NMR spectra of **L1Sn1**.*

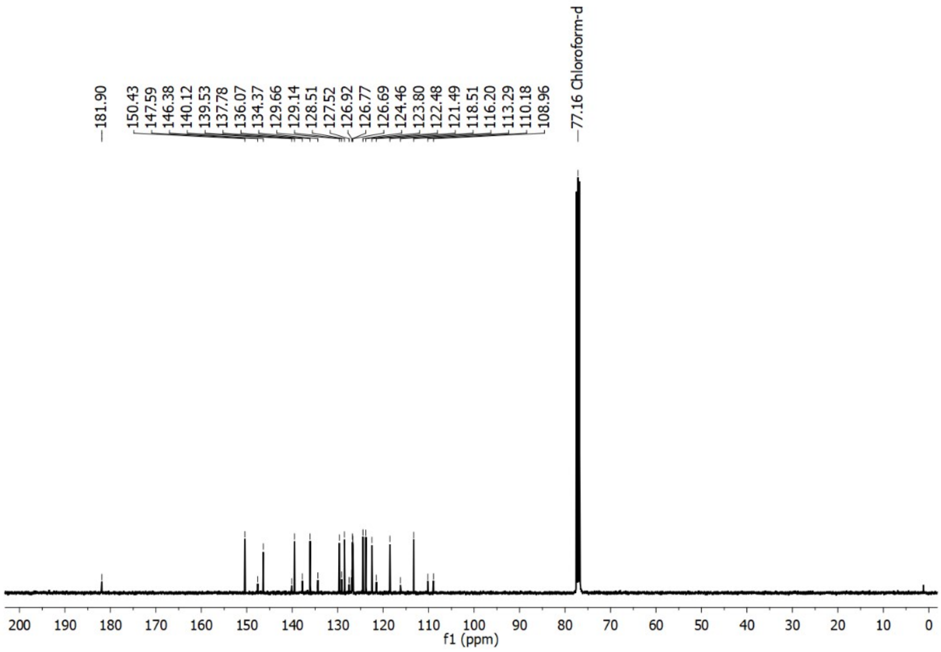

Figure S3. $^{13}C\{^{1}H\}$ *NMR spectra of **L1Sn1***.

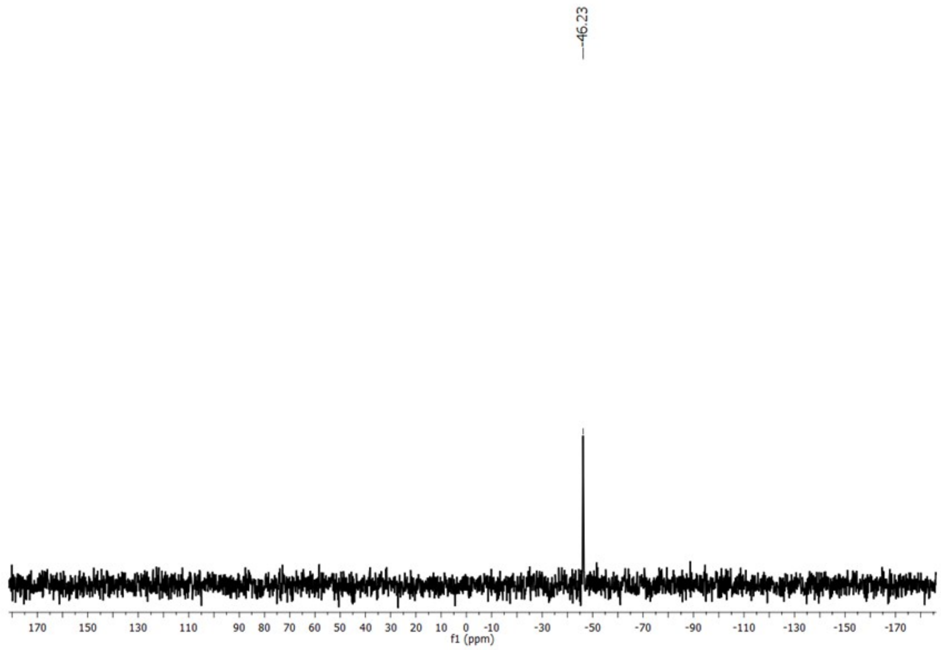

Figure S4. *^{119}Sn spectra of **L1Sn1***.

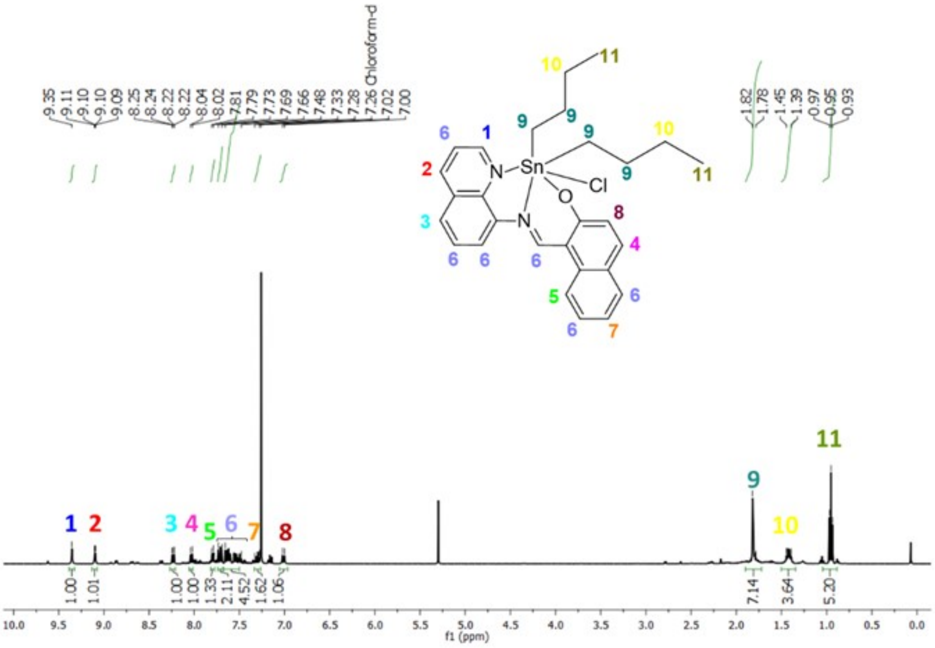

Figure S5. *^{1}H NMR spectra of* ***L1Sn2***.

Figure S6. *$^{13}C\{^1H\}$ NMR spectra of **L1Sn2**.*

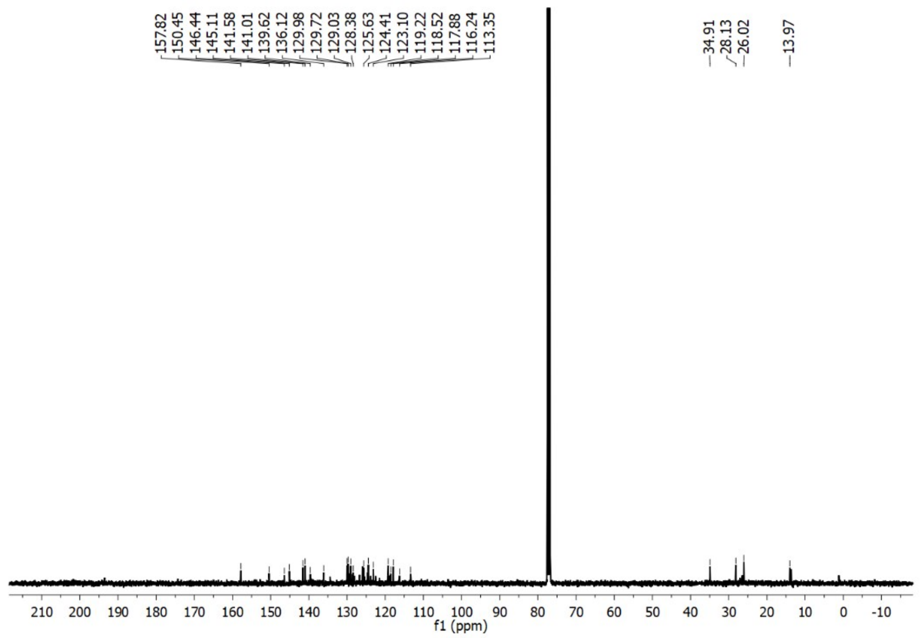

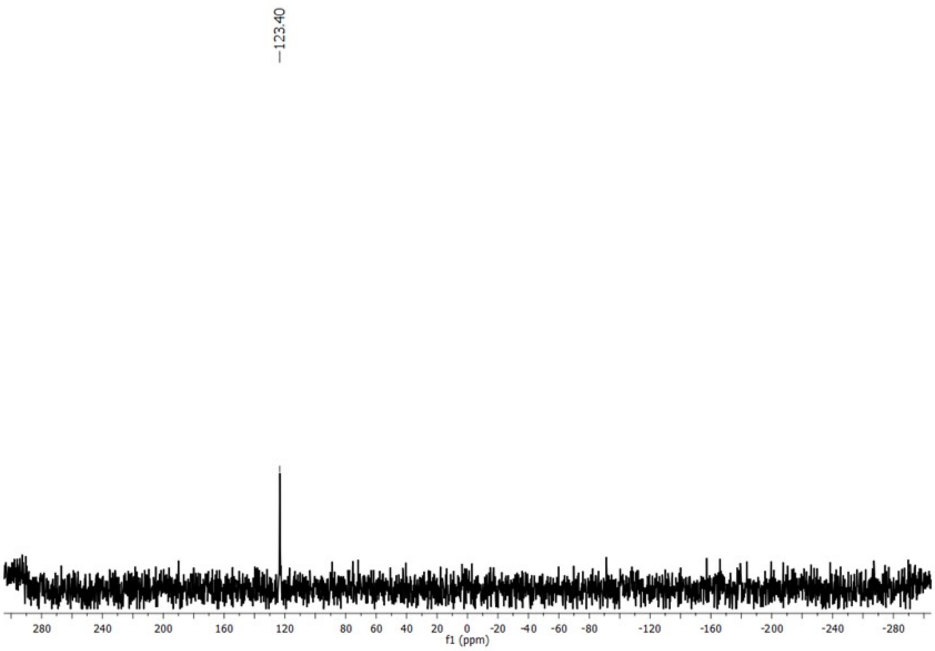

***Figure S7**. ^{119}Sn spectra of **L1Sn2**.*

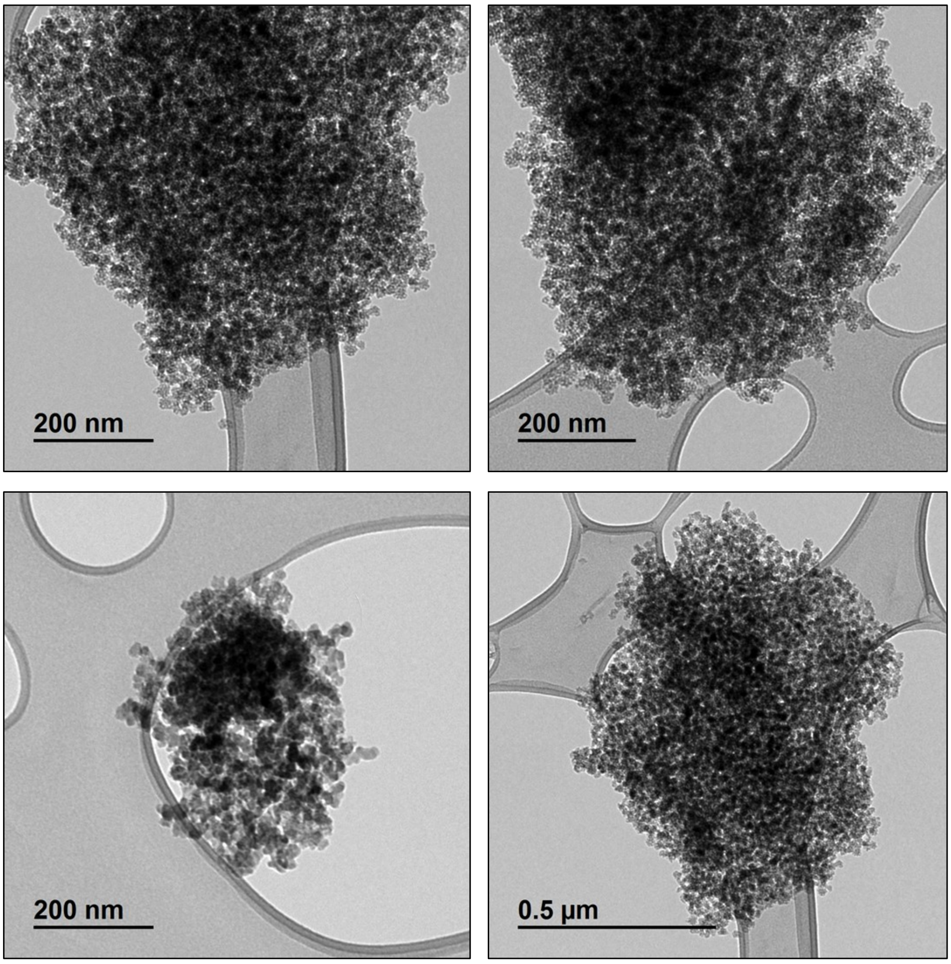

Figure S8. *TEM micrographs of starting silica material (**sMSN**).*

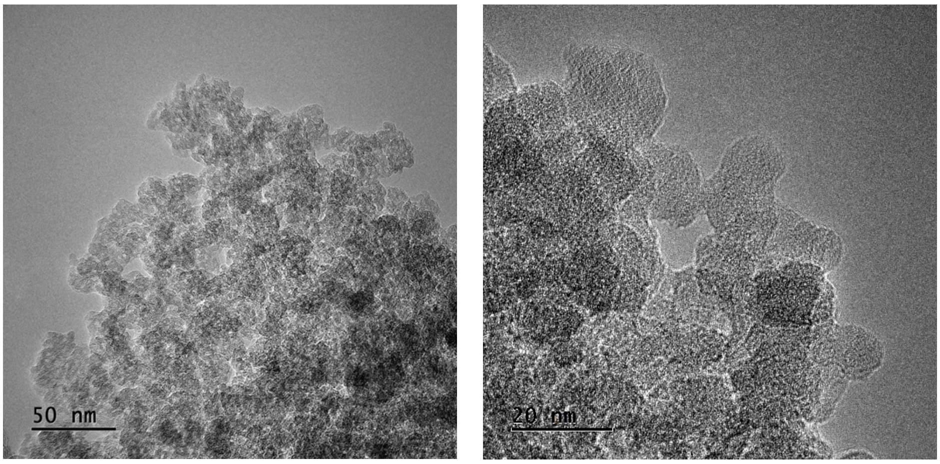

Figure S9. *STEM micrographs of the starting material* ***sMSN*** *(left) and the final material* ***sMSN-FA-L1Sn1*** *(right).*

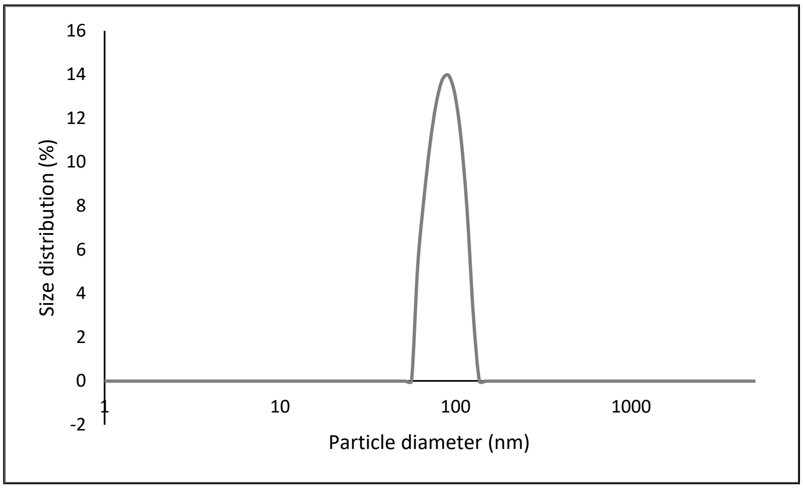

Figure S10. *Particle size of* ***sMSN*** *in PBS by dynamic light scattering.*

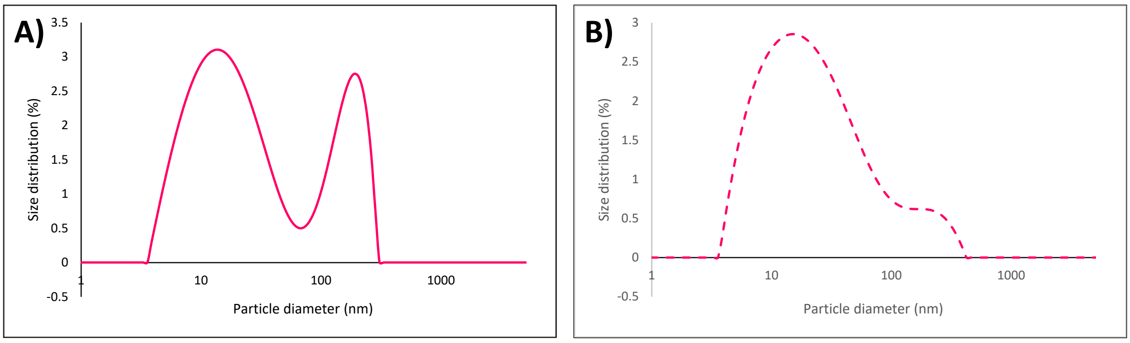

Figure S11. *Particle size of* ***sMSN*** *in DMEM by dynamic light scattering (A) and the measurement of only DMEM (B).*

Table S1. *Z-potential measurements for three final materials in PBS and DMEM media.*

	Z-potential (mV)	
	**PBS pH 7.4**	**DMEM**
**sMSN-L1Sn2**	-4.4 ± 2.5	-9.4 ± 1.8
**sMSN-FA-L1Sn2**	-4.2 ± 3.2	0.2 ± 0.5
**sMSN-BIO-L1Sn2**	-2.2 ± 1.5	-9.6 ± 3.1

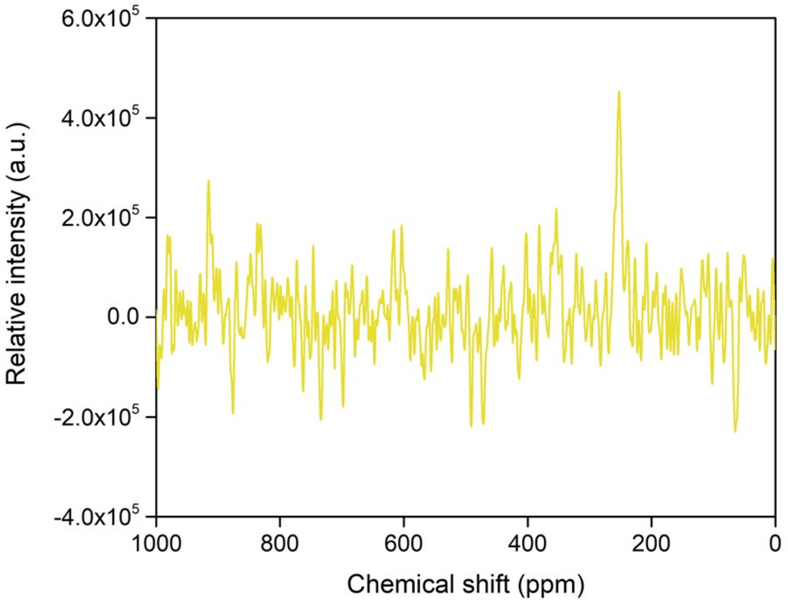

Figure S12. *^{119}Sn MAS NMR spectrum of **sMSN-L1Sn1**.*

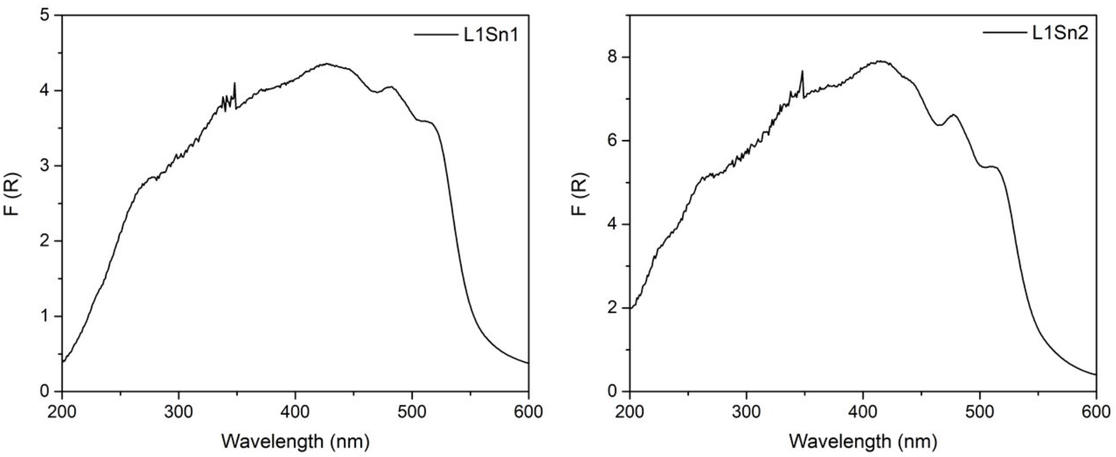

Figure S13. *Diffuse reflectance UV-visible spectra of tin complexes* ***L1Sn1*** *and* ***L1Sn2***.

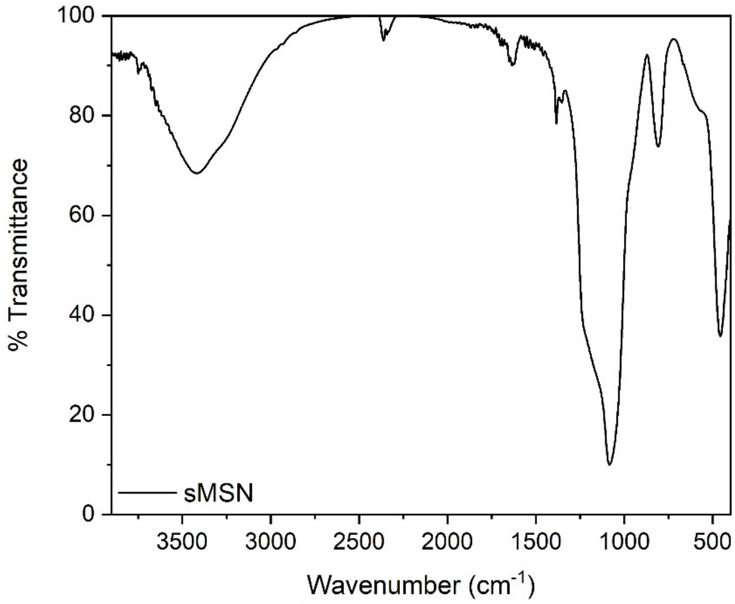

Figure S14. *FT-IR of the starting material sMSN.*

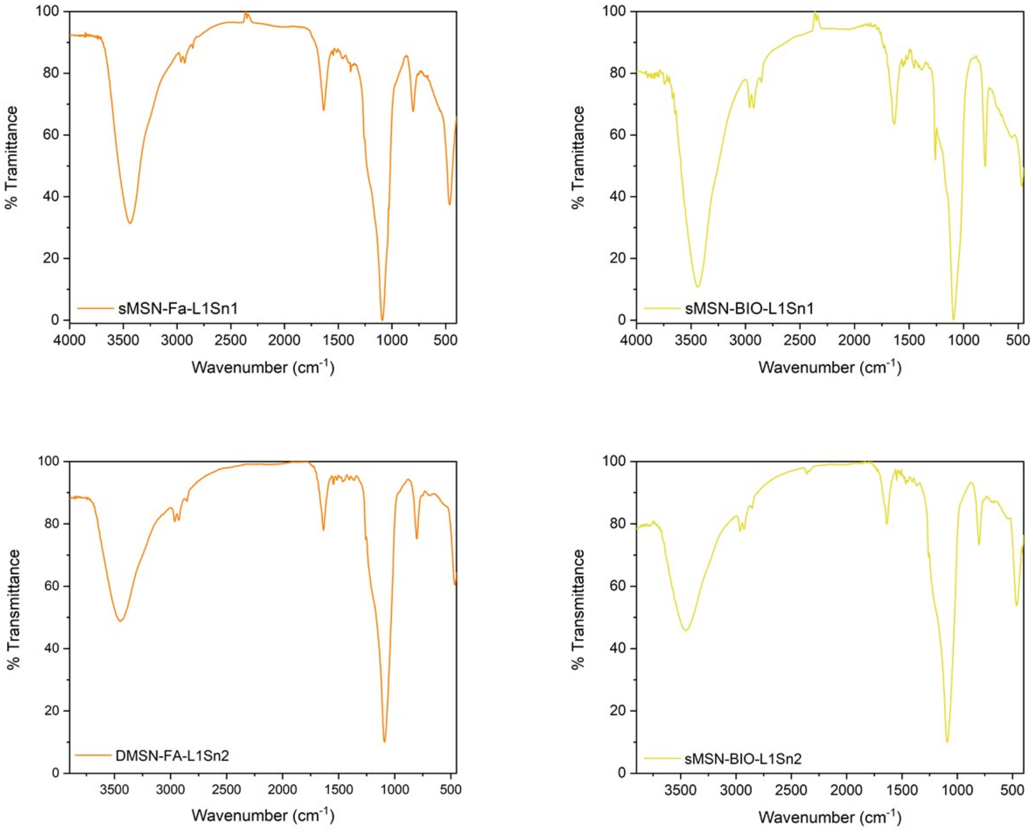

***Figure S15**. FT-IR spectra of the final materials.*

***Table S2**.Comparative tin release at different biological medium at 7 days of incubation.*

MATERIAL	%Sn release		
	PBS buffer pH 7.4	PBS buffer pH 7.4 with BSA and Glu	PBS buffer pH 5.5
**sMSN-FA-L1Sn1**	0.33	1.12	8.28
**sMSN-FA-L1Sn2**	1.00	2.74	11.43
